# Impact of COVID‐19 on the analytical diagnosing ability of family medicine residents

**DOI:** 10.1002/jgf2.393

**Published:** 2020-10-26

**Authors:** Ryuichi Ohta, Nozomi Nishikura, Chiaki Sano

**Affiliations:** ^1^ Community Care Unnan City Hospital Unnan Shimane Prefecture Japan; ^2^ Department of Community Medicine Management Faculty of Medicine Shimane University Izumo Shimane Prefecture Japan

## Abstract

This article described the difficulties of family medicine residents regarding diagnostic skill training during the pandemic of COVID‐19. Social impact may strongly affect clinical reasoning skills.

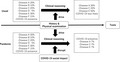

To the Editor:

Our hospital, which is in a rural area in Japan, provides community‐based medical education for undergraduate and postgraduate medical residents.^1^ Currently, in our community, the family medicine residents are fighting against the coronavirus disease (COVID‐19) pandemic on the frontline. Usually, they are trained in general wards, which accommodate patients with acute and chronic diseases and conditions, and they are orientated toward diagnosing diseases based on clinical reasoning. However, this pandemic has built a strong social norm that all patients may be potentially infected with the new coronavirus, even if asymptomatic, and those infected should be diagnosed immediately.^2^ This can disrupt the residents’ sense of pretest probability for providing differential diagnoses and change the physicians’ consideration of pretest probabilities of diseases, even in unaffected areas.

Clinical reasoning is used for disease diagnosis, consisting of System 1 (intuitive) and System 2 (analytical) thinking. As System 1 thinking depends on previous clinical experience, family medicine residents usually use System 2 thinking. They consider that the possibility of disease development is based on clinical histories and physical examinations. Thus, they make lists of possible diseases for further tests.^3^ COVID‐19 is on the list of possible diseases for all patients. Because of the pandemic, the residents were confused and gave up using System 2 thinking, which if properly applied, could help in rational clinical reasoning. Thus, the COVID‐19 pandemic affects the clinical reasoning ability of the residents (Figure 1). Our community, with a population of ~40 000 individuals, has had only three cases of COVID‐19 thus far. However, the presence of COVID‐19 has strongly affected the attitudes toward medical care.

**Figure 1:**
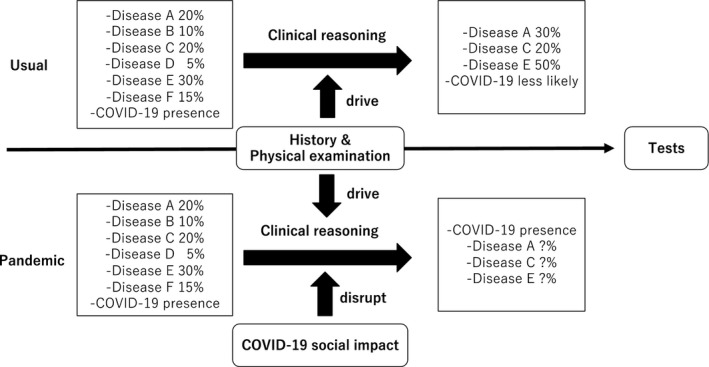
Changes in the lists of differential diagnoses based on clinical reasoning during the COVID‐19 pandemic

The clinical reasoning ability of medical professionals can be affected by experience and emotions.^4^ Generally, experienced clinicians tend to use System 1 thinking, which can be affected by their knowledge of specific diseases and change their reasoning, leading to misdiagnosis. Conversely, System 2 thinking is not affected by previous experience, thereby reducing bias and diagnostic errors. However, this pandemic has disrupted the clinical reasoning skills of family medicine residents working in our hospital (Figure 1). Our residents experienced a case wherein a patient who only had a dry cough and no contact with a COVID‐19‐infected individual was eventually diagnosed with COVID‐19 after various tests with standard precaution. Although the case was not of their patients, the public accusation against the process of diagnosis affected the residents’ differential diagnosis.

Family physicians need a comprehensive view of the diagnostic process during the COVID‐19 pandemic. Family physicians treat patients based on multiple aspects, such as the biopsychosocial model.^5^ Medical residents should be taught that social factors can change the diagnostic processes. They may unintentionally change their diagnostic processes, by, for instance, not advising invasive tests for frail, old patients. As the pandemic situation became overwhelming, they tended to be conscious regarding the change in the sensitivity of clinical reasoning. This pandemic is not only teaching us the importance of infection control but also the need for learning sociology.

## CONFLICTS OF INTEREST

Authors declare no conflict of interests for this article.
